# Complementary activity of tyrosine kinase inhibitors against secondary kit mutations in imatinib-resistant gastrointestinal stromal tumours

**DOI:** 10.1038/s41416-019-0389-6

**Published:** 2019-02-22

**Authors:** César Serrano, Adrián Mariño-Enríquez, Derrick L. Tao, Julia Ketzer, Grant Eilers, Meijun Zhu, Channing Yu, Aristotle M. Mannan, Brian P. Rubin, George D. Demetri, Chandrajit P. Raut, Ajia Presnell, Arin McKinley, Michael C. Heinrich, Jeffrey T. Czaplinski, Ewa Sicinska, Sebastian Bauer, Suzanne George, Jonathan A. Fletcher

**Affiliations:** 1 Department of Pathology, Brigham and Women’s Hospital, Harvard Medical School, 20 Shattuck Street, Thorn 528, Boston, MA USA; 2 Department of Medical Oncology, Dana-Farber Cancer Institute, Boston, MA USA; 3 Sarcoma Translational Research Laboratory, Vall d’Hebron Institute of Oncology; Department of Oncology, Vall d’Hebron University Hospital, Barcelona, Spain; 4 Department of Medical Oncology, West German Cancer Center, University Hospital Essen, University Duisburg-Essen, Essen, Germany; 5 Broad Institute of MIT and Harvard, 7 Cambridge Center, Cambridge, MA USA; 6 Department of Molecular Genetics, Lerner Research Institute and Cleveland Clinic, 9500 Euclid Ave, Cleveland, OH USA; 7 Ludwig Center for Cancer Research at Dana-Farber Cancer Institute and Harvard Medical School, Boston, MA USA; 8 Division of Surgical Oncology, Brigham and Women’s Hospital, Harvard Medical School, Boston, 75 Francis Street, Boston, MA USA; 9 Portland VA Medical Center and OHSU Knight Cancer Institute, Portland, Oregon, USA; 10 Department of Oncologic Pathology, Dana Farber Cancer Institute, Boston, MA USA; 11 Present Address: Daiichi Sankyo Inc., Basking Ridge, NJ USA

**Keywords:** Sarcoma, Predictive markers

## Abstract

**Background:**

Most patients with KIT-mutant gastrointestinal stromal tumours (GISTs) benefit from imatinib, but treatment resistance results from outgrowth of heterogeneous subclones with KIT secondary mutations. Once resistance emerges, targeting KIT with tyrosine kinase inhibitors (TKIs) sunitinib and regorafenib provides clinical benefit, albeit of limited duration.

**Methods:**

We systematically explored GIST resistance mechanisms to KIT-inhibitor TKIs that are either approved or under investigation in clinical trials: the studies draw upon GIST models and clinical trial correlative science. We subsequently modelled in vitro a rapid TKI alternation approach against subclonal heterogeneity.

**Results:**

Each of the KIT-inhibitor TKIs targets effectively only a subset of KIT secondary mutations in GIST. Regorafenib and sunitinib have complementary activity in that regorafenib primarily inhibits imatinib-resistance mutations in the activation loop, whereas sunitinib inhibits imatinib-resistance mutations in the ATP-binding pocket. We find that rapid alternation of sunitinib and regorafenib suppresses growth of polyclonal imatinib-resistant GIST more effectively than either agent as monotherapy.

**Conclusions:**

Our data highlight that heterogeneity of KIT secondary mutations is the main mechanism of tumour progression to KIT inhibitors in imatinib-resistant GIST patients. Therapeutic combinations of TKIs with complementary activity against resistant mutations may be useful to suppress growth of polyclonal imatinib-resistance in GIST.

## Background

Gastrointestinal stromal tumour (GIST) is a mesenchymal tumour of the gastrointestinal tract and the most common subtype of human sarcoma.^1,2^ KIT or PDGFRA receptor tyrosine kinase gain-of-function mutations are crucial initiating oncogenic events in 90% of GISTs,^3,4^ resulting in oncogenic addiction. Therefore, abolition of KIT or PDGFRA signalling with tyrosine kinase inhibitors (TKIs) profoundly impairs GIST cell viability and growth.^5,6^

First-line imatinib mesylate (Gleevec, Novartis Oncology, Basel, Switzerland) inhibits activity of mutant KIT and PDGFRA, and substantially improves survival in most GIST patients.^6,7^ However, most patients with initial clinical benefit from imatinib eventually progress, typically in 20–24 months.^6,7^ Oncogenically-activated KIT continues to be the key driver of GIST proliferation and survival after imatinib failure in up to 90% of the patients, due to reactivation of KIT signalling by tumour subclones with heterogeneous secondary KIT mutations.^8–11^ These KIT secondary mutations cluster in two regions of the kinase domain: the ATP-binding pocket (encoded by exons 13 and 14) and the activation loop (encoded by exons 17 and 18).

Current drug development strategies for imatinib-resistant GIST exploit continued KIT-dependency by seeking TKIs which inhibit a broader spectrum of KIT secondary mutations. These efforts led to regulatory approval of sunitinib (Sutent, Pfizer Inc.; New York, USA) and regorafenib (Stivarga, Bayer HealthCare Pharmaceuticals Inc., Montville, NJ, USA) as second- and third-line therapies, respectively, for patients with advanced GIST.^12–15^ Other multikinase inhibitors are currently in phase I to phase III clinical studies.^16,17^ Because sunitinib and regorafenib are broadly-active multi-kinase inhibitors, they are associated with more toxicities than imatinib in most GIST patients. Further, after clinical progression of advanced GIST on imatinib, the clinical benefit of sunitinib and regorafenib is limited, with median time to progression of 6 months or less.^16,17^ Our previous studies have shown that some but not all imatinib-resistant KIT-mutations respond to sunitinib. Specifically, in vitro and clinical studies demonstrated that sunitinib is active against imatinib-resistant GIST subclones with KIT ATP-binding pocket V654A secondary mutations, but inactive against subclones with KIT activation loop mutations.^12,18^ Thus, clinical progression on sunitinib occurs after a median time of 6 months,^14^ mainly due to the emergence of cross-resistant KIT-dependent subclones.^8–11^ Likewise, polyclonal heterogeneity of KIT-driven, imatinib-resistant subclones might also lead to the modest benefit observed with regorafenib and other available TKIs as single-agent therapies.

In this study we further explored the relationship between secondary KIT kinase mutations and the activity of third-line regorafenib and other TKIs with KIT inhibitory activity being investigated in GIST clinical trials. To this end, we determined the activity of multiple TKIs against imatinib-sensitive and imatinib-resistant KIT mutations in clinically representative, patient-derived GIST models. We found that regorafenib, as well as all small molecule KIT-inhibitor monotherapies, have a drug-specific activity profile against a subset of the KIT secondary mutational spectrum. Interestingly, some of these TKIs, including second- and third-line sunitinib and regorafenib, have complementary activity against resistant clones, and therefore we further undertook an in vitro proof-of-concept combination strategy based on rapid TKI alternation aiming to deter growth of polyclonal imatinib-resistant subclones.

## Materials and methods

### Cell culture studies

CHO cells were from the American Type Culture Collection (Manassus, VA, USA) and were transfected with *KIT*-mutant cDNA constructs as previously described^9^ and treated with imatinib, sunitinib or regorafenib. GIST cells used in these studies were derived from human GIST metastases and have been published previously^19^ with the exception of GIST226, which is a novel KIT-negative GIST line that contains (but does not express) homozygous primary *KIT* exon 11 in-frame deletion (P551-W557) and homozygous *KIT* exon 17 Y823D mutations. All lines were credentialed by Sanger sequencing evaluations of known mutations, at baseline and every 3 months during the study. All cultures were shown to be mycoplasma-free.

### Protein blotting

Whole cell lysates were prepared as described previously,^20^ and protein concentrations were determined using the Bio-Rad protein assay (Bio-Rad, Hercules, CA, USA). KIT immunoprecipitations, in the CHO cell assays, were as described previously.^9^ Electrophoresis, immunoblotting, and chemiluminescence detection were as described previously.^21^ Primary antibodies to phospho-KIT Y721 (#3391), phospho-KIT Y703 (#3073), phospho-AKT S473 (#9271), AKT (#9272), phospho-RB1 S795 (#9301) and RB1 (#9309) were from Cell Signaling Technology (Danvers, MA, USA); to KIT (#A4502) were from Dako (Carpinteria, CA, USA); to actin (#A4700) were from Sigma (San Luis, MI, USA); and to Cyclin A (clone 6E6) were from Leica Byosistems (Wetzlar, Germany).

### Immunohistochemistry

Immunohistochemical staining for Ki-67 was performed against cell cultures on chamber slides with an antibody (#0505) from Immunotech (Marseille, France) at dilution of 1:200. Then the slides were incubated with a biotin-conjugated secondary antibody and stained using the Ventana (Tucson, AZ, USA) DAB detection kit with counterstaining by haematoxylin.

### Reagents

Ponatinib and regorafenib were from Selleck Chemicals (Houston, TX, USA). Dovitinib, dasatinib, imatinib, masitinib, nilotinib, sunitinib, and sorafenib were from LC Laboratories (Woburn, MA, USA).

### Cell viability studies

The sulforhodamine B (SRB) assay was used according to the method of Skehan.^22^ Cells were plated in 96-well flat-bottomed plates. After 24 h culture medium was replaced with fresh medium (with or without drugs) in triplicate cultures. At the end of drug exposure (72 h), cells were fixed for 1 h and stained with 0.4% SRB (Sigma Aldrich, St. Louis, MO USA) and the optical density was detected at 560 nm. Each experiment was repeated three times.

### Clinical correlative studies

Tumour specimens for genotype analyses were obtained from patients enrolled on a phase II clinical trial of regorafenib in GIST.^23^ Briefly, patients were adults who had histologically confirmed metastatic and/or unresectable GIST with progression or intolerance to imatinib and prior failure to sunitinib. Tumour tissue was analysed in patients receiving regorafenib 160 mg daily 3-weeks on, 1-week off. Objective response was assessed by computed tomography (CT) in genotyped patients at baseline and at the end of every even-numbered cycle. Disease status was assessed using Response Evaluation Criteria in Solid Tumours (RECIST) as complete response (CR), partial response (PR), stable disease (SD), or progressive disease (PD).^24^ Metabolic response was assessed by serial [^18^F]fluoro-2-deoxy-D-glucose positron emission tomography (FDG-PET) scans were done in a fasting state 1 h following i.v. administration of FDG (15–20 mCi) at baseline, at the end of cycle 1 and cycle 4 dosing.

### GIST xenograft studies

A patient-derived xenograft (PDX) model, PG48, was developed from the regorafenib-resistant GIST patient #1. This PDX has a homozygous *KIT* exon 11 primary mutation (V559D) and a homozygous *KIT* exon 13 secondary ATP-binding pocket mutation (V654A). All in vivo work was conducted under appropriate Institutional Animal Care and Use-Committee-approved protocols. Six- to 8-week-old female adult athymic nude mice (NMRI nu/nu) were obtained from Charles River Laboratories (Wilmington, MA, USA) and housed under specific pathogen-free conditions. Tissue fragments of PG48 were serially passaged in donor mice injected subcutaneously in each rear flank. In all studies, vehicle control or study drugs were administered orally once daily. Solutions and drug doses were as follows: sterile water, and 100 mg/kg/day for Imatinib; citrate buffered (pH 3.5), and 40 mg/kg/day for sunitinib^25^; PEG400/125 mM aqueous methanesulphonic acid (80/20), and 30 mg/kg/day for regorafenib.^15^ The experiment was stopped after 3 days of treatment, mice were sacrificed, and tumours were harvested for protein analysis.

### Drug-withdrawal studies

GIST cell lines were cultured in serum-containing media in the presence of DMSO, imatinib, sunitinib or regorafenib at the indicated concentrations. Drugs were withdrawn and washed out, and cells were grown in regular media. Study time-points were as follows: day 0 (on drug), and days 1, 3 and 7 after drug withdrawal. Cultures were performed in 6-well plates to obtain cell lysates to assess KIT pathway and cell cycle activation, and in Lab-TEK II chamber slides (Thermo Fisher, Walthman, MA, USA; #154526) to assess cell proliferation (KI67 immunostaining and mitotic count) as described above.

### Validation of a rapid-alternation schedule in mixed GIST cell cultures

Human GIST cells with clinically representative KIT mutations (GIST-T1, GIST430/654 and GIST-T1/820) were stably infected with lentiviruses containing specific 24-basepair DNA “barcodes”, as described previously.^26^ Equally numbers of each of the three cell lines were added to create mixed GIST cell cultures. Mixed GIST cell cultures were treated with sunitinib (200 nM) and regorafenib (400 nM), as single-agents or in rapid-alternation. Genomic DNA was isolated using QIAamp DNA Mini Kit (Quiagen, Germantown, MD, USA) on day 0 (baseline), 7, 14, 21 and 28, and analysed using PRISM technology.^27^ PRISM allows assessment of relative cell viability in mixtures of cell lines via the quantification of unique DNA barcodes incorporated into individual cell lines. Results are shown as percentage of cell numbers relative to untreated. Two-sided unpaired t-test is used for cell number comparisons between treatment conditions.

## Results

### Each of nine small molecule KIT-inhibitors has activity against only a subset of common imatinib-resistance oncogenic KIT mutations

Nine TKIs that have either been approved or are under clinical investigation as KIT-inhibitors for GIST were evaluated by viability assays in GIST cell lines representing four biologic categories: 1) imatinib-sensitive with KIT primary mutations; 2) imatinib-resistant due to KIT ATP-binding pocket secondary mutations; 3) imatinib-resistant due to KIT activation loop secondary mutations; and 4) imatinib-resistant with acquired loss of KIT oncoprotein expression (acquired KIT-negative). Each of these TKIs was active against GIST cells with *KIT* primary mutations in exon 11 (GIST-T1 and GIST430) but failed to inhibit the full range of secondary imatinib-resistance mutations (Table 1). Imatinib and masitinib were ineffective against all of the common KIT secondary mutations. The other seven TKIs had activity against GIST cells with some but not all imatinib-resistance mutations (Table 1). Sunitinib and dovitinib inhibited viability of GIST cells dependent on the *KIT* exon 13 V654A secondary mutation, with IC_50_ values lower than 500 nM (IC_50_ 45 nM and 250 nM, respectively). The ponatinib IC_50_ for GIST with KIT V654A was 100 nM, but this exceeds the clinically relevant concentration (<28 nM) for this drug,^28^ confirming previous evidence that ponatinib is not sufficiently active against this common resistance mutation.^19^ A broader group of TKIs (sunitinib, regorafenib, sorafenib, ponatinib and dovitinib) inhibited GIST cells dependent on the “gatekeeper” *KIT* exon 14 T670I mutation (IC_50_ 5–200 nM). Sunitinib and dovitinib lacked activity against secondary mutations in the KIT activation loop (*KIT* exons 17 and 18), in contrast to regorafenib, sorafenib, nilotinib, ponatinib and dasatinib, which inhibited viability of GIST cells dependent on some or all of the activation-loop mutants (Table 1). No substantial TKI effects were observed in KIT-independent GIST cell lines GIST48B and GIST226 (Table 1), which underscores that TKI-activity is typically mediated by blocking KIT signalling in imatinib-resistant GIST. These observations are consistent with previously reported clinicopathologic evidence that short-duration clinical responses in KIT-mutant GIST, after development of imatinib-resistance, result from outgrowth of cross-resistant GIST subpopulations with both ATP-binding pocket and activation loop KIT secondary mutations.^8–11^

**Table 1:** Cell viability IC50 values for nine KIT-inhibitor TKIs in GIST cell lines

GIST CELL LINE	KIT MUTATION	IMATINIB	SUNITINIB	REGORAFENIB	SORAFENIB	NILOTINIB	PONATINIB	MASITINIB	DASATINIB	DOVITINIB
GIST-T1	Ex 11	4.5	5	35	30	15	4	15	4	4
GIST430	Ex 11	35	10	150	40	50	5	30	10	50
GIST882	Ex 13	300	70	800	300	350	50	350	150	500
GIST430/654	Ex 11 + Ex 13 (V654A)	2500	45	2000	800	850	100	3500	1000	250
GIST-T1/670	Ex 11 + Ex 14 (T670I)	>10,000	30	60	150	5000	5	10000	n.r.	200
GIST-T1/816	Ex 11 + Ex 17 (D816E)	1500	>10,000	550	650	500	40	6500	300	6000
GIST-T1/820	Ex 11 + Ex 17 (D820A)	1500	>10,000	600	600	400	25	3000	90	1200
GIST-T1/829	Ex 11 + Ex 18 (A829P)	3000	10000	2500	1500	650	25	>10,000	70	1500
GIST48B	KIT-independent	>10,000	>10,000	>10,000	>10,000	>10,000	1500	>10,000	>10,000	750
GIST226	KIT-independent	>10,000	>10,000	>10,000	>10,000	8000	6000	>10,000	>10,000	>10,000

### Regorafenib activity in GIST is mediated through KIT oncogenic signalling inhibition and displays a complementary activity profile with sunitinib against KIT secondary mutations

**Fig. 1:**
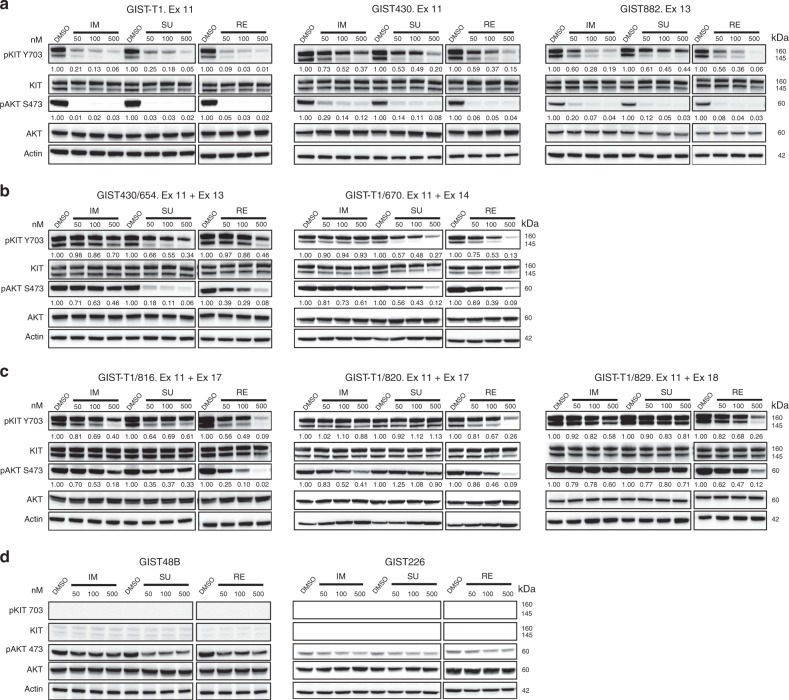
KIT oncoproteins in GIST are differentially inhibited by sunitinib and regorafenib. Immunoblotting evaluations of phospho-KIT and downstream phospho-AKT were performed in GISTs belonging to four clinical-genotypic categories: (**a**) imatinib-sensitive GISTs contained only KIT primary mutations; (**b**) imatinib-resistant GISTs with ATP-binding pocket KIT secondary mutations; (**c**) imatinib-resistant GISTs with activation loop KIT secondary mutations; (**d**) KIT-negative GISTs. This figure also provides quantifications, relative to the DMSO-only controls (normalised to 1.0), of the (**a**–**c**) phosphoKIT and phosphoAKT responses in KIT-dependent GISTs

Evaluations of the three FDA- and EMA-approved drugs for the treatment of GIST, imatinib, sunitinib and regorafenib, demonstrated that each drug inhibited oncogenic KIT and downstream AKT phosphorylation in a dose-dependent manner in GIST cells containing only *KIT* primary exon 11 mutations (GIST-T1, GIST430 and GIST882) without *KIT* secondary mutations (Fig. 1a). By contrast, in GIST cells with *KIT* exon 11 primary mutations coupled to common secondary *KIT* imatinib-resistance mutations, KIT and downstream signalling were variably inhibited by sunitinib and regorafenib. Specifically, GIST cells in which *KIT* exon 11 primary mutation was coupled to a secondary mutation in exon 13 V654A mutation were inhibited only by sunitinib, whereas GIST cells with *KIT* exon 11 primary mutation coupled to an exon 14 T670I gatekeeper mutation were inhibited by sunitinib and regorafenib (Fig. 1b), and GIST cells with *KIT* exon 11 primary mutation coupled to exon 17 D816E or D820A or exon 18 A829P activation loop mutations were inhibited by regorafenib only (Fig. 1c). Neither sunitinib nor regorafenib inhibited viability or AKT phosphorylation in KIT-negative GIST cell lines GIST48B and GIST226 (Fig. 1d). Consistent with these in vitro results in GIST cells, evaluations of primary and secondary *KIT* mutants transfected in Chinese Hamster Ovarian (CHO) cells showed that sunitinib was more active than regorafenib against the *KIT* exon 13 V654A ATP-binding pocket mutant, whereas regorafenib was more active than sunitinib against *KIT* exon 17 activation loop mutants (Supplementary Figure 1).

### Correlative science studies show suboptimal regorafenib clinical activity against the common KIT V654A imatinib-resistance mutation in GIST

**Fig. 2:**
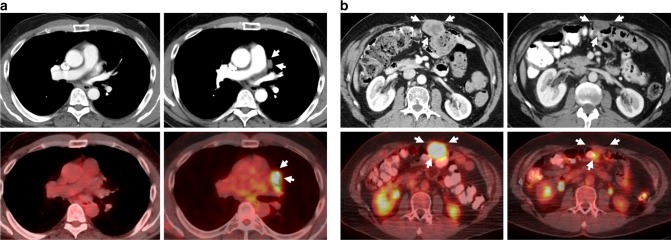
Clinical evidence that GIST imatinib-resistant KIT mutations have differential responsiveness to regorafenib. Two GIST patients were treated at the standard regorafenib dose (160 mg/d, 3 weeks on, 1 week off) and imaging and tissue specimens were available. **a** Patent #1 developed a new site of metastatic disease after 12 cycles of treatment, which was FDG-avid in the PET/CT. Resection and sequencing of this progressing lesion showed a V654A KIT ATP-binding pocket secondary mutation. **b** Patient #2 had a 4.5 × 3.2 cm metastatic lesion in the abdominal wall that was biopsied and sequenced prior to regorafenib therapy. A KIT activation-loop imatinib-resistance mutation in exon 17 (D820Y) was found. After 5.5 months on regorafenib treatment, tumour size diminished to 3.0 × 1.6 cm and SUV values decreased from 9.9 to < 3

Imaging studies and tissue biopsies were obtained from two GIST patients treated with regorafenib in a phase II clinical trial.^23^ These two patients received standard regorafenib dosing (160 mg, 3-weeks on, 1-week off) throughout the study. After 9 months on therapy, patient #1 developed a metabolically active mediastinal lesion (Fig. 2a), which was resected. Histologically, the lesion lacked treatment effect, and genotype analysis revealed a primary *KIT* exon 11 mutation and a secondary *KIT* exon 13 V654A mutation. The patient resumed regorafenib treatment and, after 12 months, developed further resistant disease in the abdomen; analysis of this lesion again revealed a *KIT* exon 13 V654A mutation, in addition to the primary *KIT* exon 11 mutation. By contrast, patient #2 had pre-treatment biopsy demonstrating primary *KIT* exon 11 and secondary D820Y (exon 17) mutations and achieved complete metabolic response and partial response by RECIST after 4 cycles of treatment with regorafenib (Fig. 2b). Complete metabolic response in this lesion was seen 18 days after treatment initiation.

We extended these in vitro and clinical data in a xenograft model derived from a regorafenib-resistant GIST, which contained *KIT* exon 11 and *KIT* exon 13 (V654A) mutations. In this model, KIT phosphorylation was inhibited substantially (80%) by sunitinib treatment, but not (8%) by regorafenib treatment (Supplementary Figure 2).

### Proof-of-concept of rapid TKI alternation treatment in heterogeneous GIST co-cultures

**Fig. 3:**
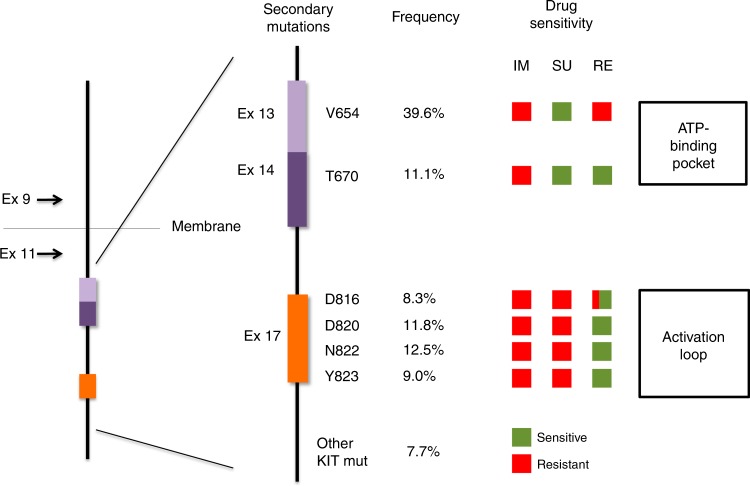
Schematic view of representative KIT secondary mutations after imatinib failure, frequency according to prior reports,^8,9,11,18,36,37^ and predicted activity profile of the three drugs currently approved for the treatment of GIST based on our studies. Green and red denotes sensitive and resistant, respectively, to imatinib (IM), sunitinib (SU) and regorafenib (RE). Regorafenib square for D816 is both red and green due to the presence of resistant amino acid changes (i.e., D816V is highly resistant to all TKIs)

Because the above-mentioned in vitro*,* in vivo and clinical data indicated complementary activity of sunitinib and regorafenib against the most common KIT imatinib-resistance secondary mutations (Fig. 3), we hypothesised that combined treatment with sunitinib and regorafenib might suppress a broad range of resistant subclones in GIST patients progressing on imatinib. However, sunitinib and regorafenib share overlapping toxic effects, and concurrent treatment with these drugs would likely exacerbate each drug’s toxicities. Therefore, we modelled a concept for rapid alternation of these drugs, aiming to inhibit growth of heterogeneous cross-resistant subclones, while minimising toxicity. Because this alternating dosing could permit regrowth of targeted subclones when the relevant drug for those subclones is withheld, we evaluated a rational time-frame for drug withdrawal, to establish a treatment schedule that minimises GIST regrowth during alternating drug withdrawal periods.

**Fig. 4:**
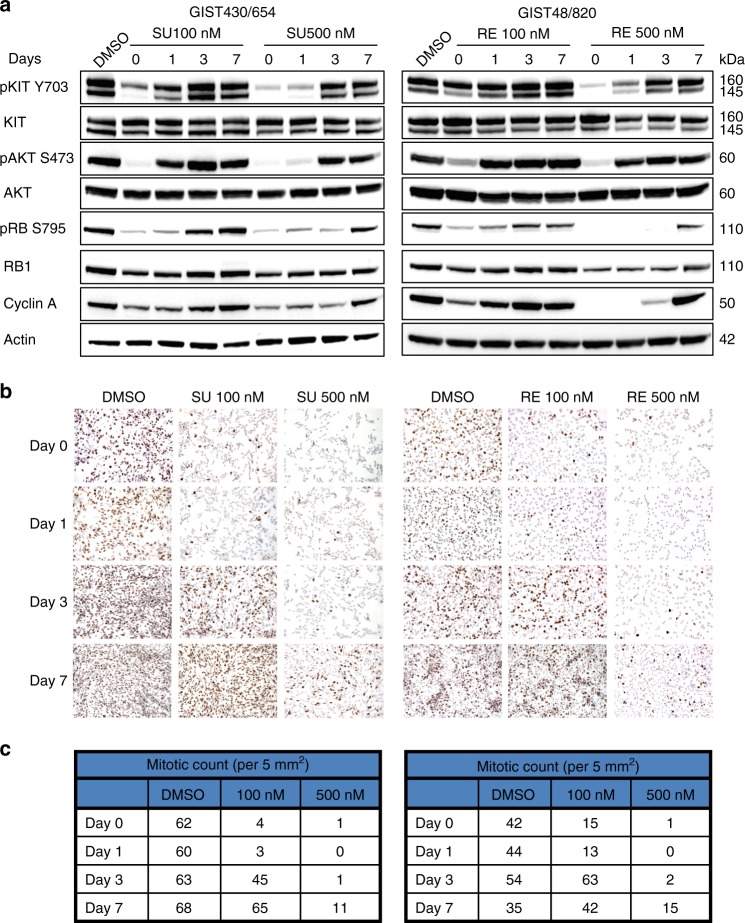
Restoration of GIST oncogenic signalling pathways and proliferation after TKI withdrawal. **a** Immunoblotting evaluations show reactivation of KIT and AKT, as assessed by phospho-KIT and phospho-AKT, and show reactivation of the cell cycle, as assessed by phospho-RB1 and Cyclin A. **b** Ki-67 staining shows recovery of proliferation after drug withdrawal. **c**, Mitotic counts (per 5 mm^2^) show recovery of proliferation after drug withdrawal

Drug withdrawal studies were performed using sunitinib-sensitive GIST430/654 (Table 1) and regorafenib-sensitive GIST48/820,^19^ after treatment with sunitinib and regorafenib, respectively. In these studies, the drugs were withdrawn after inhibition of KIT phosphorylation and cell proliferation was achieved. Partial re-activation of KIT phosphorylation and downstream AKT phosphorylation was observed 24 h after drug withdrawal in both cell lines. Cell cycle re-activation, as evidenced by upregulation of phospho-RB1, cyclin A, and Ki-67, occurred 3- to- 7 days after drug withdrawal (Fig. 4a). Complete restoration of Ki-67 expression and mitotic activity occurred 7 days after drug withdrawal (Fig. 4b and c). These effects on KIT signalling and cell cycle re-activation were dose-dependent. These observations define a time-frame for TKI alternation in which substantial recovery of in vitro cell proliferation occurs within 7 days, whereas minimal, if any, recovery is seen within 3 days after TKI withdrawal.

**Fig. 5:**
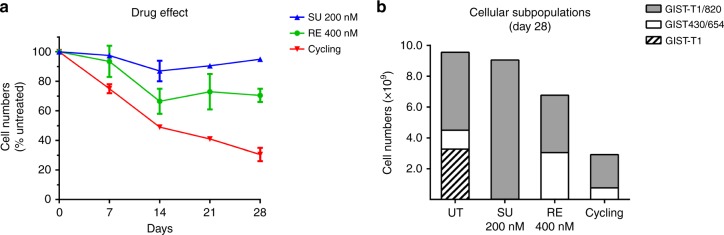
Suppression of polyclonal imatinib-resistant populations by sunitinib and regorafenib rapid alternation. PRISM analysis of barcoded cell lines was used to assess mixed GIST cultures treated with single-agent sunitinib, single-agent regorafenib, or rapid alternation (3 days of sunitinib alternating with 4 days of regorafenib). **a** Cell numbers were lowest in the rapid alternation arm, at all time-points. **b** Population profiling at day 28 demonstrated partial suppression of ATP-binding-pocket (V654A) and activation loop (D820A) imatinib-resistant GIST cells by the rapid alternation approach

To test the effects of drug withdrawal and rapid drug alternation on heterogeneous GIST populations, we established polyclonal co-cultures of GIST cell lines containing clinically relevant *KIT* mutations: exon 11 mutation alone (GIST-T1), exon 11 primary mutation *in cis* with exon 13 secondary mutation (GIST430/654), and exon 11 primary mutation *in cis* with exon 17 secondary mutation (GIST-T1/820), modelling the polyclonal imatinib-resistance heterogeneity observed in many GIST patients. Each cell line was labelled with a unique DNA barcode to allow quantification of the relative amount of each line in the co-culture using the PRISM method.^27^ Co-cultures were established with equal representation of the three cell lines and expanded for 4 weeks under the following conditions: (1) untreated; (2) continuous sunitinib; (3) continuous regorafenib; or (4) rapid alternation of sunitinib and regorafenib (sunitinib 3 days, alternating with regorafenib 4 days). The sunitinib and regorafenib alternation was more effective than either drug alone with respect to inhibiting growth of these polyclonal populations and preventing emergence of a single dominant clone (Fig. 5a). In the GIST polyclonal co-cultures receiving monotherapies, we observed overrepresentation of the known cross-resistant clone. Although imatinib-resistant co-cultures were not eradicated, total numbers of both GIST-T1/820 and GIST430/654 were reduced in the co-cultures treated with rapid alternating combination of sunitinib and regorafenib (Fig. 5b).

## Discussion

The crucial oncogenic events in most GISTs are gain-of-function KIT mutations, which cause KIT tyrosine kinase constitutive activation and resultant constitutive signalling through growth-promoting pathways, including PI3K/AKT/mTOR and RAS/RAF/MEK.^29^ These primary KIT mutations typically involve *KIT* exons 9 or 11, corresponding to extracellular dimerization domains and intracellular inhibitory juxtamembrane domains, respectively. The imatinib-resistance mechanisms responsible for clinical progression in KIT-mutant GISTs commonly involve emergence of polyclonal subpopulations with secondary KIT kinase domain mutations.^8–11^ KIT-independent mechanisms leading to imatinib-progression in KIT-mutant GIST have been reported but are infrequent and yet to be thoroughly studied.^17,30^ Therefore, KIT inhibitory strategies after failure of imatinib remain useful because most imatinib-resistant GISTs are still dependent upon KIT signalling for survival and proliferation. This has been evidenced by GIST antiproliferative responses in both preclinical models and patients, after treatment of imatinib-resistant GISTs by various KIT-inhibitor strategies, including RNA interference,^9^ alternative KIT kinase inhibitors such as sunitinib,^12,18^ or HSP90 inhibitors.^31^ However, the frequently polyclonal nature of GIST imatinib-resistance constitutes a clinical challenge and explains why sunitinib, the current second-line standard-of-care, generally has only modest efficacy in GIST patients.^14^ Conceivably, this heterogeneous nature of KIT secondary mutations also limits the clinical benefit of subsequent lines of treatment, including third-line regorafenib^13^ and other TKIs under investigation, such that median time to progression is in the same range as that with sunitinib.^16,17^

We and others have previously demonstrated that sunitinib clinical activity for most GISTs is determined by the primary and secondary KIT mutations in those tumours.^12,18^ In the present study, we provide the first evidence of regorafenib spectrum of inhibition of imatinib-resistant KIT mutations. We profiled regorafenib activity against a panel of GIST cell lines containing the common types of KIT imatinib-resistance secondary mutations in GIST, involving either the KIT ATP-binding pocket or activation loop. Regorafenib, like imatinib and sunitinib, had compelling activity against GIST models dependent only on *KIT* exon 11 primary mutations, i.e. lacking imatinib-resistant secondary *KIT* mutations. In addition, regorafenib inhibited KIT kinase activation loop secondary imatinib-resistance mutations, which are known to be sunitinib resistant.^12,18^ The evidence for regorafenib activity against KIT secondary activation loop mutations included preclinical modelling of drug effects on cell proliferation, KIT phosphorylation, and KIT-pathway phosphorylation. Further, we document regorafenib clinical response in a patient whose GIST had a KIT activation loop imatinib-resistance mutation (Fig. 2b), supporting evidence we reported previously.^23^ Nonetheless, KIT activation loop secondary mutations are heterogeneous and the present study demonstrates variable regorafenib activity depending on the exact activation loop amino acid alteration. For example, regorafenib was more active against *KIT* exon 17 D816E mutation than D816H. Other KIT oncogenic mutations, including an exon 13 K642E primary mutation, and the exon 14 T670I “gatekeeper” secondary mutation, were inhibited effectively by both sunitinib and regorafenib.^32,33^ Very similar GIST inhibition profiles were demonstrated for regorafenib and the structurally-related compound, sorafenib, demonstrating the reproducibility of the models.

Regorafenib had only modest activity against the *KIT* exon 13 V654A secondary mutation, which modifies a residue in the ATP-binding pocket of the kinase and is the most common secondary imatinib-resistance mutation in GIST patients following primary treatment with imatinib.^18^ In the present study, a patient progressed twice on a standard regorafenib dose/schedule, and on each occasion the regorafenib-resistance resulted from KIT V654A secondary mutation. Likewise, in preclinical studies, we demonstrated modest inhibition of KIT V654A phosphorylation only at high doses of regorafenib. Therefore, the evidence to date indicates that regorafenib does not inhibit effectively the *KIT* V654A imatinib-resistance mutation in GIST. By contrast, KIT V654A is inhibited effectively by sunitinib, as shown in our present evaluations and in previously published preclinical and clinical studies.^12,18^

Our cell viability studies using 9 different TKIs, either approved or under clinical investigation in GIST, underscore that each of the targeted KIT-inhibitor small molecule drugs included in this analysis is active against only a subset of the common imatinib-resistance secondary KIT mutations. As discussed above, these limitations have been established in previous studies for sunitinib,^12,18^ and are now reported, herein, for regorafenib. Likewise, our current studies confirm that ponatinib inhibits some but not all common imatinib-resistance secondary KIT mutations, with the common *KIT* exon 13 V654A mutation being a particular challenge.^19^ Although the drug screens show differential activity for each of the TKIs against various imatinib-resistance KIT mutations, the in vitro IC_50_ values do not permit precise prediction of clinical activity, particularly in absence of correlative studies from GIST biopsies and/or cfDNA in patients receiving these agents. Overall, regorafenib and sorafenib had comparable activity in vitro. Although sorafenib IC_50_ for *KIT* exon 13 V654A mutation appears to be lower compared to regorafenib (800 nM vs. 2000 nM), we do not expect meaningful clinical differences for these two drugs. Masitinib activity fully overlaps with imatinib, showing a >10–100-fold gap between IC_50_ in imatinib-sensitive vs. imatinib-resistant mutants. Nilotinib and dasatinib have similar profiles, with activity in imatinib-sensitive models and potential activity against activation-loop mutants, which agrees with previous reports.^34,35^ However, and in the light of published clinical data in imatinib-resistant disease, it is unlikely that nilotinib and dasatinib activity against KIT secondary mutations in the activation loop is comparable to that of regorafenib or ponatinib.^16,17^ Finally, dovitinib activity appeared to be comparable to sunitinib in our studies, with the IC_50_ for ATP-binding pocket mutations being 6–30-fold lower than those for mutations in the activation-loop. All studies in KIT-independent cell lines confirm that the activity of these TKIs is mediated through KIT oncogenic signalling inhibition. Together, these studies reinforce that KIT secondary genotype determines the activity of TKIs with KIT inhibitory activity in GIST, thereby establishing the molecular basis for the modest clinical benefit observed with successive lines of treatment in imatinib-resistant GIST.

The incomplete coverage of imatinib-resistant KIT mutations by each of these drugs allows cross-resistant GIST subclones to emerge from a background of heterogeneous GIST cells with varied KIT secondary mutations, leading to clinical progression irrespective of the TKI used. Therefore, more effective strategies for KIT-inhibition are needed in patients with imatinib-resistant GIST, in order to accomplish longer-lasting clinical benefit. Hence, we investigated whether rational combinations of KIT inhibitors could overcome polyclonal resistance after imatinib failure. Notably, several drugs from our screening displayed complementary activity profiles with respect to inhibiting either the *KIT* exon 13 V654A mutation or *KIT* exon 17 activation loop mutations. Each of these mutation categories accounts for approximately 40% of imatinib-resistant subclones in GIST patients (Fig. 3).^8–10,18,36,37^ In order to enable translation into a near-term clinical trial in GIST patients, we focused in these preclinical studies on novel approaches to administering sunitinib and regorafenib, the two currently approved drugs for imatinib-resistant GIST. Therapeutic combinations may augment the magnitude and/or duration of clinical responses,^38,39^ and the observed complementary pattern suggests that a combination of sunitinib and regorafenib could broaden the spectrum of subclones effectively targeted by second-line therapy, delaying the emergence of cross-resistant disease and extending the window of clinical benefit beyond the 4–6 months currently observed with each drug as single-agent.^13,14^ However, both drugs share overlapping toxicities and concurrent treatment would require dose reductions leading to loss of efficacy in order to minimise adverse events.^38,39^ Several studies have shown that continuous dosing is not necessary for drug efficacy,^40–44^ and might decrease selective pressure and minimise positive subclonal selection and subsequent disease progression:^41,42^ these observations beg the question as to whether sequentially alternating sunitinib and regorafenib could achieve effective doses of both drugs while minimising toxicity. Thus, we identified and validated in GIST cell models a concept for rapid-alternation of sunitinib and regorafenib, aiming to maintain inhibitory pressure on the two major types of imatinib-resistance KIT mutations that coexist in many GIST patients. These data provide rationale for a clinical trial in which alternation of sunitinib for 3 days with regorafenib for 4 days is currently being evaluated in GIST patients with advanced disease (ClinicalTrials identifier: NCT02164240).

In summary, this study defines the spectrum of inhibition of imatinib-resistant KIT mutations by third-line regorafenib and multiple available TKIs. We find that small molecule KIT-inhibitor monotherapies have drug-specific activity profiles against only subsets of the KIT secondary imatinib-resistance mutations, which constitutes the molecular basis for the modest clinical benefit observed with successive lines of treatment in imatinib-resistant GIST. Our studies underscore the need for therapies that suppress the spectrum of heterogeneous imatinib-resistant subclones that can arise in a given GIST patient. Leveraging these insights, we show that rapid alternation of TKIs with complementary activity can control heterogeneous imatinib-resistance subclones.

## Supplementary information

Supplementary Figure 1

Supplementary Figure 2

## Data Availability

All the conclusions reached by the authors are supported by available main and supplementary tables and figures.
