# Correction: Complementary activity of tyrosine kinase inhibitors against secondary kit mutations in imatinib-resistant gastrointestinal stromal tumours

**DOI:** 10.1038/s41416-019-0487-5

**Published:** 2019-05-23

**Authors:** César Serrano, Adrián Mariño-Enríquez, Derrick L. Tao, Julia Ketzer, Grant Eilers, Meijun Zhu, Channing Yu, Aristotle M. Mannan, Brian P. Rubin, George D. Demetri, Chandrajit P. Raut, Ajia Presnell, Arin McKinley, Michael C. Heinrich, Jeffrey T. Czaplinski, Ewa Sicinska, Sebastian Bauer, Suzanne George, Jonathan A. Fletcher

**Affiliations:** 1 Department of Pathology, Brigham and Women’s Hospital, Harvard Medical School, 20 Shattuck Street, Thorn 528, Boston, MA USA; 2 Department of Medical Oncology, Dana-Farber Cancer Institute, Boston, MA USA; 3 Sarcoma Translational Research Laboratory, Vall d’Hebron Institute of Oncology; Department of Oncology, Vall d’Hebron University Hospital, Barcelona, Spain; 4 Department of Medical Oncology, West German Cancer Center, University Hospital Essen, University Duisburg-Essen, Essen, Germany; 5 Broad Institute of MIT and Harvard, 7 Cambridge Center, Cambridge, MA USA; 6 Department of Molecular Genetics, Lerner Research Institute and Cleveland Clinic, 9500 Euclid Ave, Cleveland, OH USA; 7 Ludwig Center for Cancer Research at Dana-Farber Cancer Institute and Harvard Medical School, Boston, MA USA; 8 Division of Surgical Oncology, Brigham and Women’s Hospital, Harvard Medical School, Boston, 75 Francis Street, Boston, MA USA; 9 Portland VA Medical Center and OHSU Knight Cancer Institute, Portland, Oregon USA; 10 Department of Oncologic Pathology, Dana Farber Cancer Institute, Boston, MA USA; 11 Present Address: Daiichi Sankyo Inc., Basking Ridge, NJ USA

**Keywords:** Sarcoma, Predictive markers

**Correction to:** *British Journal of Cancer* (2019) **120**, 612–620; 10.1038/s41416-019-0389-6; published online 22 February 2019.

The additional information of this manuscript originally stated that the authors declare no competing interests. This statement was incorrect, and should instead have stated the following:

M.C.H. has the following competing interests to declare: Equity interest at Molecular MD; Consulting at Molecular MD, Blueprint Medicines, Deciphera Pharmaceuticals; Expert Testimony at Novartis; Licensed patent with royalty payments at Novartis. The remaining authors have no competing interests to declare.

The authors apologise for any convenience this may have caused.

