# Supplementary figures and images for: Complementary activity of tyrosine kinase inhibitors against secondary kit mutations in imatinib-resistant gastrointestinal stromal tumours
Source: Br J Cancer. 2019 Feb 22;120(6):612–20. doi: 10.1038/s41416-019-0389-6 (PMC6462042; doi:10.1038/s41416-019-0389-6)

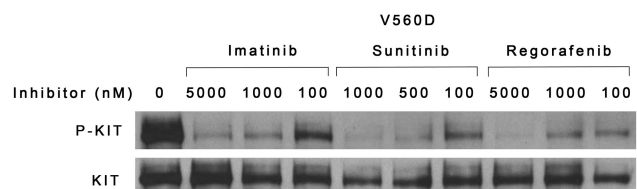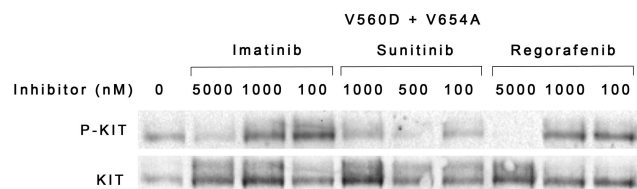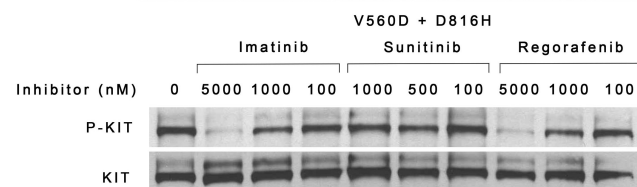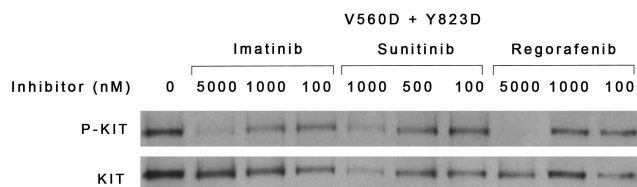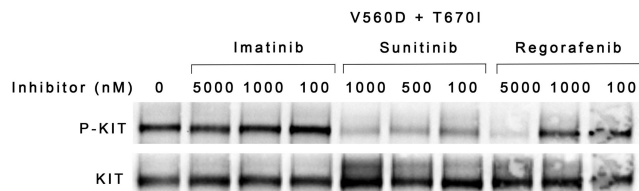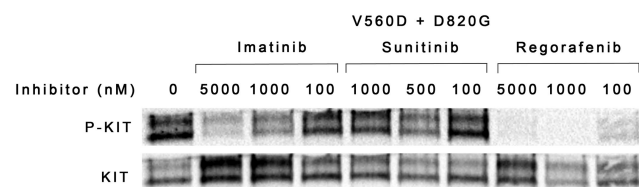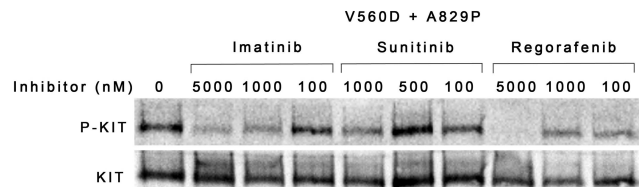

Supplement: Supplementary file 1 — Supplementary Figure 1 [file 41416_2019_389_MOESM1_ESM.pdf]
