# Supplementary material for: Complementary activity of tyrosine kinase inhibitors against secondary kit mutations in imatinib-resistant gastrointestinal stromal tumours
Source: Br J Cancer. 2019 Feb 22;120(6):612–20. doi: 10.1038/s41416-019-0389-6 (PMC6462042; doi:10.1038/s41416-019-0389-6)
Supplement: Supplementary file 2 — Supplementary Figure 2 [file 41416_2019_389_MOESM2_ESM.pdf]

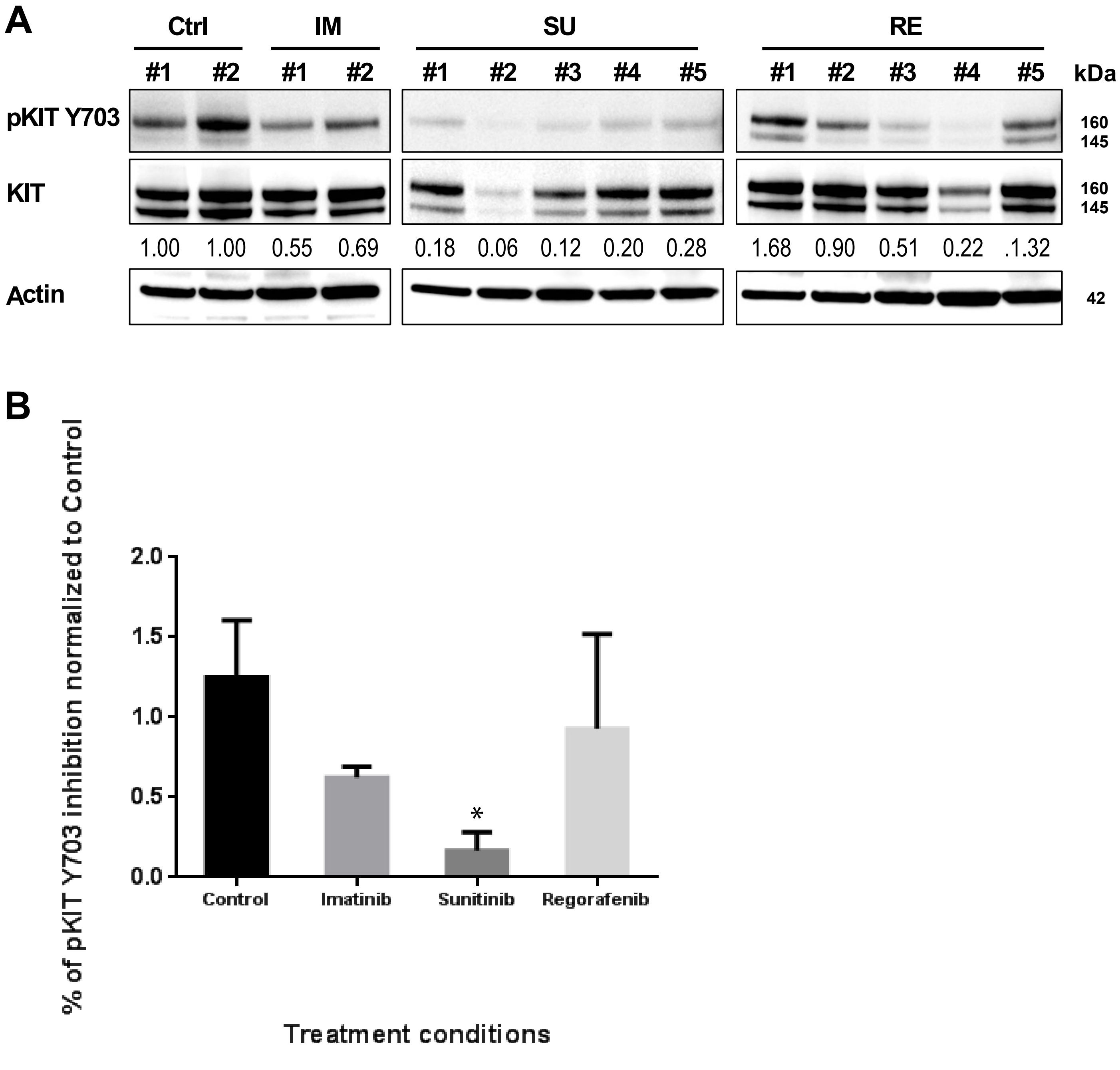

Supplementary Figure 3. A. Comparative activity of imatinib (IM), sunitinib (SU) and regorafenib (RE) vs. untreated control (CTRL) using PG48 GIST PDX with homozygous KIT exon 11 (V559D) and exon 13 (V654A) mutations. Mice were treated with drugs for 3 days. **B.** Immunoblotting phospho-KIT quantifications are relative to the normalized average of the two controls. Total actin is a loading control.
